# Exploring the Antecedents of Money Attitudes in China: Evidence From University Students

**DOI:** 10.3389/fpsyg.2022.888980

**Published:** 2022-06-01

**Authors:** Yuqian Li, Fengfei Hu

**Affiliations:** ^1^School of Teacher Education, Nanjing Xiaozhuang University, Nanjing, China; ^2^School of Physical Education, Nanjing Normal University, Nanjing, China

**Keywords:** money, belief in *guanxi*, need for power, need for achievement, China

## Abstract

With rapid economic growth and institutional reform, the pursuit of money and material possessions has become the most prevalent value in contemporary China. This study focuses on the cultural root of money attitudes among the young adults. Specifically, 332 Chinese university students participated in a survey to report on their need for power, need for achievement, belief in *guanxi*, and love of money. Confirmatory factor analysis and regression analysis were applied to test the proposed hypotheses. The results show positive influences of need for power and need for achievement on individuals’ love of money. Moreover, belief in *guanxi* mediates the relationship between need for power and love of money. The application of indigenous cultural concepts in analyzing social behavior in Eastern cultures is emphasized. Limitations and directions for future research are also discussed.

## Introduction

The great economic growth and institutional reform in the past four decades have substantially changed social norms and individuals’ behaviors in China. Modern China has witnessed a drastic transition from “to be poor is glorious” in the pre-opening up era to collective pursuit of wealth and material possessions (Faure and Fang, 2008; Yang and Stening, 2013). Leung (2008) states that “materialism” is the most fitting term to describe contemporary Chinese culture.

As the essence of industrialized society, money is the core instrument of business and the measure of value (Smith, 1776/1937). In a collectivistic culture such as China’s, people are very likely to be affected by others; thus, there is a tendency to conform to uniform values and chase common goals (Sun et al., 2014b). In the pre-opening up era, the prevalent goals were “serving the people wholeheartedly” and “dying for the interests of the country” (Sun et al., 2014a). Reform initiatives since 1978 have held economic growth as the first priority and insisted that all efforts should serve this goal. The government proposed a pragmatic strategy that focuses on concrete goals such as developing productivity and raising people’s living standards. Under these circumstances, “it is glorious to be rich” has permeated all of society as a collective ideology (Faure and Fang, 2008). Today, money has become the most prevalent topic in the daily lives of Chinese people.

Love of money reflects one’s attitudes toward money. It affects various social and managerial behaviors, such as pay and job satisfaction, turnover intention, and unethical behavior (e.g., Tang et al., 2000; Tang and Chiu, 2003; Froese and Xiao, 2012). However, few studies investigate its antecedents, especially from cultural perspectives. This research focuses on money attitudes among Chinese university students and explores their antecedents from an indigenous *guanxi* perspective. We believe cultural constructs developed by Western societies may fail to completely capture the nuances in Eastern society. Indigenous concepts could help researchers explain specific phenomena in a particular culture and extend that understanding to social behavior (Chinese Culture Connection, 1987).

China is a hierarchical society with a high power distance (Hofstede, 1980) and strict ordering relationships (Bond and Hwang, 1986). Under these circumstances, people are very sensitive to their positions in the social structure (Leung and Chan, 2003). Cheng (1990) argued that the social role, not the self, determines one’s behavior. Gao et al. (1996) found that in China, the impact of an individual’s voice heavily depends on his or her social position. When an individual has obtained a high standing in society, they are more likely to possess substantial control over scarce resources. Thus, in China, people are keen to attain achievement and power that can advance the ultimate goal to obtain wealth and social approval. From a young age, Chinese people are told, “You must work hard to get ahead of others.” This indoctrinates people to compete with others to gain achievements and status rather than settle for a self-contented life. For an adult, the major criterion for selecting one’s job is income, not personal interest. This is also reflected in consumer culture. Branded products are bought to signify power, achievement, and status rather than to satisfy personal needs (Li et al., 2015; Sun et al., 2016; Wang et al., 2020).

In addition, Confucianism contends that society is based on the five cardinal relationships (*wulun*): the relationships between ruler and subjects, parent and child, husband and wife, elder and younger sibling, and friends. A Chinese man would view himself as a son, a father, a husband, a brother, and a leader. In these relationships, everyone should behave properly according to his or her own position. Chinese people believe this is the ideal way to organize a harmonious society through a well-defined hierarchy, so they accept this established structure and tend to respect and defer to authority (King and Bond, 1985).

*Guanxi*, a term that literally means “interpersonal connections,” originated in this cultural context. It is regarded as a cultural orientation that reflects a series of traditional ethical concepts, such as hierarchy, interdependence, and reciprocity (Hwang, 1987). Chinese people need to deal with *guanxi*-related issues in their everyday lives. To build *guanxi*, first people are required to avoid conflict and maintain harmonious relationships with others (Leung et al., 2011). However, conflict avoidance alone is far from enough. Quality *guanxi* further requires the maintenance of long-term relationships based on mutual commitment, obligation, and especially benefit. Because people with high social positions take advantage in resource distribution, having good *guanxi* with power and authority can help one obtain psychological and practical benefits. Previous studies have highlighted the profound implications of *guanxi* for business activities in China. For instance, in a workplace, high-quality *guanxi* with supervisors (Cheung et al., 2009; Li et al., 2018), customers (Li et al., 2017), business partners, and government authorities (Gu et al., 2008; Bedford, 2011; Chen and Bedford, 2021) greatly improves job satisfaction and work performance from the personal to the corporate level. In this study, we identify *guanxi* as a cultural root of people’s love of money in China and explore its role in predicting individuals’ love of money.

### Need for Power, Need for Achievement, and Love of Money

Achievement and power are basic human needs (Schwartz, 1992, 1994). In modern society, money is the key indicator that signifies achievement and power. Thus, people with a high need for achievement and power tend to satisfy their needs through the pursuit of money. In modern industrialized societies, people prefer to consider themselves independent from others. Individual attitudes are the dominant factor influencing their behavior, and the social normative influence is weak; thus, the pursuit of money is mainly due to a desire for personal achievement. However, in China, such a traditionally collectivistic country with a high power distance, people rely on others to live and are very concerned about their social position and prestige. It is believed that traditional and modernized mindsets co-exist in contemporary China (Faure and Fang, 2008). Thus, we propose that both need for power and need for achievement drive individuals’ love of money, and we in turn propose the following hypotheses:

H1a: Need for power positively relates to love of money.
H1b: Need for achievement positively relates to love of money.

### The Mediating Role of Belief in *Guanxi*

*Guanxi* reflects “an informal, particularistic personal connection between two individuals who are bounded by an implicit psychological contract” (Chen and Chen, 2004, p. 306). It emphasizes some personal qualities, such as loyalty, obedience, and sincerity (Tan and Snell, 2002). In the Chinese organizational environment, *guanxi* determines one’s prospects to a large extent (Bozionelos and Wang, 2006; Wei et al., 2010). At the corporate level, it can lower transaction costs (Standifird and Marshall, 2000), substitute for formal legal structure (Xin and Pearce, 1996), and/or provide a competitive advantage that helps companies achieve superior performance (Luo and Chen, 1997; Tsang, 1998). Thus, it is identified as the core competence leading to success in China for both individuals and organizations (Yeung and Tung, 1996; Zhang and Zhang, 2006; Gu et al., 2008).

In previous studies, researchers have classified *guanxi* into different types (Hwang, 1987; Tsang, 1998; Su and Littlefield, 2001; Fan, 2002a,b; Chen and Chen, 2004; Zhang and Zhang, 2006). First, *qinqingguanxi* is present among family members or relatives (*jiaren*) based on blood and driven by emotional affection and obligations. This type of *guanxi* is strong and stable, so it could last for a long time. Second, *renqingguanxi* is based on favorable exchanges. The participants in *renqingguanxi* are mostly acquaintances (*shuren*), such as friends, classmates, and colleagues. In this type of *guanxi*, the participants need to observe the reciprocity rule, or they will owe others a debt (*renqingzhai*). The last category is *jiaoyi guanxi*. This type of *guanxi* is highly related to rent-seeking and power abuse. When people have difficulties, they tend to first ask for help from powerful people who can use social dominance to obtain benefits on their behalf. It is believed some *renqingguanxi* and most *jiaoyi guanxi* happens in workplace and business environments, essentially through the process of exchanging money for power and vice versa (*quanqianjiaoyi*) (Su et al., 2003). Fan (2002a,b) maintains that business (*jiaoyi*) *guanxi* links money to power and corruption. He further asserts that all business *guanxi* is tainted by corruption, and every act of corruption is related to using *guanxi*. Similarly, Lovett et al. (1999) even considers *guanxi* synonymous with corruption and bribery.

Luo (2008) proposes a taxonomy of intertwinement between *guanxi* and power based on two dimensions: the form of *guanxi* and the level of power abuse. According to Chinese tradition, people should not ask for money when they offer help, or else it will impair affection (*tan qianshangganqing*). Even if you want to thank the helper, a small gift is enough to express your deep affection (*li qingyizhong*). Otherwise, the *guanxi* will be damaged (*jianwai*).

However, when materialism and money worship become prevalent in society, people judge success primarily based on material possessions and monetary wealth. Under these circumstances, *guanxi* between *jiaren* and *shuren* also has an instrumental character that relates to unethical behavior. Thus, some researchers have expressed concern that the rampant use of *guanxi* for personal gain denies social justice and leads to a morally bankrupt society (Fan, 2002a; Luo, 2008). Because *guanxi* greatly relates to power and money in Chinese society, positive relationships between these concepts are expected. Specifically, people who seek power tend to use *guanxi* to obtain monetary benefits. Considering the strong correlation between power and achievement, we assert that *guanxi* links achievement to money as well. In this study, we define belief in *guanxi* as the extent to which an individual thinks *guanxi* can help one achieve business success, we propose it will play a mediating role in the relationship between individuals’ need for power/achievement and love of money, and we in turn propose the following hypotheses:

H2a: Belief in *guanxi* mediates the relationship between need for power and love of money.
H2b: Belief in *guanxi* mediates the relationship between need for achievement and love of money.

## Materials and Methods

For this study, data were collected from students of a public university in Jiangsu Province, East China. We distributed the survey and collected the data online. A cover letter was attached to ensure that participation was voluntary and anonymous. A total of 332 students participated in this research, 129 males and 203 females. The mean age of the sample population was 20.3 years.

### Measures

Established scales are used to measure the variables investigated in this study. Specifically, we adopt the scales from Liu et al. (2010) to measure need for power and need for achievement. Each construct is measured with four items. Sample items include “I want other people to act in my way,” and “I love to confront the challenges of the job.” The Cronbach’s α for need for power and need for achievement are 0.83 and 0.83, respectively.

Six items were selected from Su et al. (2003) to measure the degree to which *guanxi* is important in business activities. A sample item is “In business, it is important to maintain a good network of relationships.” The Cronbach’s α is 0.91.

Four items were adopted from Tang and Chiu (2003) to measure love of money. A sample item is “Money is important.” The Cronbach’s α is 0.92.

All the items were rated with a 7-point Likert scale. The translation and back-translation methods were used.

### Data Analysis

Before examining the hypothesized relationships, we first conducted a confirmatory factor analysis (CFA) with AMOS to estimate the measurement model, which consists of four constructs measured by 18 observed items. In Table 1, the CFA result of the baseline four-factor model was compared with those of three alternative models. The results indicate that the alternative models exhibited significantly poorer fit than the baseline model, which shows a fairly good fit (Steiger, 1980): χ^2^ = 245.7, *df* = 129, χ^2^/*df* = 1.90, GFI = 0.93, CFI = 0.97, TLI = 0.96, RMSEA = 0.05. Moreover, all factor loadings are above the critical value of 0.5 (ranging from 0.63 to 0.92) for adequate individual item reliability (Bagozzi and Yi, 1988). In addition, the composite reliabilities of all the scales are all greater than 0.8 (ranging from 0.83 to 0.92), which illustrates sound psychometric properties (Bagozzi and Yi, 1988). The average variance extracted (AVE) for each of the four constructs is above 0.54.

**TABLE 1:** Confirmatory factor analysis of the measurement models.

Model specifications	χ^2^	*df*	GFI	CFI	TLI	RMSEA
Baseline four-factor model: NFP, NFA, BIG, LOV	245.7	129	0.93	0.97	0.96	0.05
Three-factor model: NFP + NFA, BIG, LOV	598.7	132	0.80	0.87	0.85	0.10
Two-factor model: NFP + NFA, BIG + LOV	1367.7	134	0.62	0.65	0.60	0.17
Four constructs represent a single dimension	1848.8	135	0.54	0.51	0.45	0.20

*NFP, need for power; NFA, need for achievement; BIG, belief in guanxi; LOV, love of money.*

Table 2 shows that no correlation between any two variables exceeds the square root of their AVE, demonstrating adequate discriminant validity between each construct and any other construct (Fornell and Larcker, 1981). Specifically, need for power positively relates to need for achievement (*r* = 0.37, *p* < 0.01), belief in *guanxi* (*r* = 0.42, *p* < 0.01), and love of money (*r* = 0.29, *p* < 0.01). Belief in *guanxi* weakly correlates with need for achievement (*r* = 0.16, *p* < 0.01) but has a stronger relationship with need for power (*r* = 0.48, *p* < 0.01).

**TABLE 2:** Correlations between the variables.

	1	2	3	4
1. Need for power	0.74^a^			
2. Need for achievement	0.37^**^	0.75		
3. Belief in *guanxi*	0.42^**^	0.16^**^	0.79	
4. Love of money	0.29^**^	0.21^**^	0.48^**^	0.86

** p < 0.01.

*^a^Numbers on the diagonal show the square root of the AVE.*

We follow the steps suggested by Baron and Kenny (1986) to test the mediating effect. A series of regression analyses was conducted with SPSS. The results of model 2 in Table 3 show that after controlling for age and gender, both need for power (*r* = 0.25, *p* < 0.01) and need for achievement (*r* = 0.13, *p* < 0.05) positively affect love of money. When we included belief in *guanxi* in model 3, the influence of need for power on love of money was not significant. However, need for achievement was still correlated with love of money (*r* = 0.13, *p* < 0.05), and the magnitude of effect did not change. Moreover, the increased *R*^2^ value (0.16) resulting from adding belief in *guanxi* in the regression is relatively large, illustrating that the impact of need for power on love of money was completely mediated by belief in *guanxi* (Baron and Kenny, 1986). Thus, H1a, H1b, and H2a are supported, but H2b is not validated.

**TABLE 3:** Results of the regression analyses.

Variables	Love of money		
Model 1	Model 2	Model 3
**Control variables**			
Age	–0.01	0.01	–0.00
Gender	–0.02	0.02	0.05
**Independent variable**			
Need for power		0.25**	0.06
Need for achievement		0.13*	0.13*
**Mediator**			
Belief in *guanxi*			0.45**
*R* ^2^	0.00	0.10	0.26
*ΔR* ^2^	0.00	0.10	0.16

*p < 0.05; **p < 0.01.

## Discussion

Its strong economic development and huge population make China fertile ground for both business leaders and social science researchers. We are witnessing an increasing number of studies focusing on this ancient but vigorous market. However, its unique culture, history, institutions, and other socio-economic factors present a challenge.

This study explores the mechanism by which the need for power and achievement drives money orientation among Chinese university students. Specifically, belief in *guanxi* mediates the influence of need for power on love of money, but it does not play the same role in the relationship between need for achievement and love of money. This result shows that although achievement and power are strongly correlated, they are distinct from each other. Power refers to social prestige, control, or dominance over people and resources, whereas achievement is defined as personal success through demonstrating competence (Schwartz, 1992, 1994). Subtle differences can be found in the definitions. Achievement is individual-directed and based on personal effort, whereas power is social-directed and mostly manifested in the process of interactions with others. This can explain why, compared to achievement, power is more closely related to *guanxi*, which originated in Eastern collectivistic societies.

In addition, this finding validates the proposition that *guanxi* carries additional connotations of power and rent-seeking that facilitate the achievement of monetary success. Ubiquitous materialism leads to pervasive pragmatism: almost everything is used as a means to pursue the ultimate goal—money. This attitude alters the meaning and significance of things. For example, it is well known that the Chinese place great importance on education. The real reason for this is that educational success can pave the way to power and authority, which is the major means of accessing scarce resources (Lin and Wang, 2010). MBA or EMBA courses have become a venue where students can build a *guanxi* network (Faure and Fang, 2008). Some business schools even set up golf classes because *guanxi* is not only built at the dinner table but also on the golf course.

### Implications

As Asia’s economies develop and its emerging markets become the focus of business research, unique cultural insights are required (Burgess and Steenkamp, 2006). Indigenous concepts could help researchers explain specific phenomena in a particular culture. For instance, an important traditional Confucian concept, face (*mianzi*), has already been identified as a major factor that drives brand name fever among Asian consumers (Wang et al., 2019; Zhang and Wang, 2019; Sun et al., 2021). Researchers have also examined its roles in consumers’ responses during service encounters (Keh and Sun, 2008; Chan et al., 2009). Other Chinese indigenous variables, such as harmony (*hexie*) and social favors (*renqing*), also have great business implications (Yen et al., 2011; Sun et al., 2014a; Li et al., 2017). Indigenous constructs from other societies include the Korean concept of *cheong*, reflecting human affection (Choi et al., 1993) and the Mexican concept of *simpatia*, which means avoidance of conflict (Triandis et al., 1984).

Although these concepts originated in non-Western societies, they are not necessarily culture specific. In fact, Western scholars studied face several decades ago (Goffman, 1955, 1967). Western people also try to gain face, so the concept does exist in Western societies (Ho, 1976; Ting-Toomey and Kurogi, 1998). Recent empirical cross-cultural studies have found evidence of the face construct in both Eastern and Western respondents (Cocroft and Ting-Toomey, 1994; Oetzel and Ting-Toomey, 2003; Chan et al., 2009). Similarly, belief in fate (*karma*), originating from Buddhism, is popular in China and India and affects consumer behavior (Chan et al., 2009; Kopalle et al., 2010), but it is also found in other societies as a universal dimension (Leung et al., 2002). An interesting study shows that a series of Chinese indigenous personality dimensions—including face, harmony, social favor, and defensiveness (*AhQ* attitude)—are replicated fairly well in an American sample, demonstrating the generalizability of these indigenous constructs (Lin and Church, 2004). This idea provides a new possibility for testing the role of culture in business and social behavior in the future: start with initial research on a question in one’s own culture based on indigenous concepts and then test the theory in other cultures. If possible, make a cross-cultural comparison to build a cultural-universal theory. This proposition is consistent with Berry’s (1989) five-step imposed etic process.

### Limitations and Directions for Future Research

The current study has several limitations. First, our sample only includes university students. It has been shown that values and mindsets differ across generational cohorts that have totally different childhood experience (Ralston et al., 1999; Hung et al., 2007; Wang et al., 2022). In the future, scholars should replicate this research in other populations, and, if possible, make a cross-generational comparison to enhance generalizability. Second, because this is a cross-sectional study, we could not verify the existence of causal relationships. In future, longitudinal or experimental research should be conducted to further validate the findings.

## Data Availability Statement

The raw data supporting the conclusions of this article will be made available by the authors, without undue reservation.

## Ethics Statement

Ethical review and approval was not required for the study on human participants in accordance with the local legislation and institutional requirements. Written informed consent for participation was not required for this study in accordance with the national legislation and the institutional requirements.

## Author Contributions

YL and FH developed the theoretical framework and completed the manuscript writing. YL worked on data collection and analysis. Both authors contributed to the article and approved the submitted version.

## Conflict of Interest

The authors declare that the research was conducted in the absence of any commercial or financial relationships that could be construed as a potential conflict of interest.

## Publisher’s Note

All claims expressed in this article are solely those of the authors and do not necessarily represent those of their affiliated organizations, or those of the publisher, the editors and the reviewers. Any product that may be evaluated in this article, or claim that may be made by its manufacturer, is not guaranteed or endorsed by the publisher.
